# Cerebrospinal Fluid IL-17A Could Predict Acute Disease Severity in Non-NMDA-Receptor Autoimmune Encephalitis

**DOI:** 10.3389/fimmu.2021.673021

**Published:** 2021-05-13

**Authors:** Michael Levraut, Véronique Bourg, Nicolas Capet, Adrien Delourme, Jérôme Honnorat, Pierre Thomas, Christine Lebrun-Frenay

**Affiliations:** ^1^ URRIS, Unité de Recherche Clinique Cote d’Azur-UR2CA, CRCSEP, Hôpital Pasteur 2, Centre Hospitalier Universitaire de Nice, Nice, France; ^2^ Internal Medicine Department, Hôpital l’Archet 1, Centre Hospitalier Universitaire de Nice, Nice, France; ^3^ Neurology Department, Hôpital Pasteur 2, Centre Hospitalier Universitaire de Nice, Nice, France; ^4^ French Reference Center for Paraneoplastic Neurological Syndromes and Autoimmune Encephalitis, Hospices Civils de Lyon, Hôpital Neurologique, Lyon, France; ^5^ Synatac Team, NeuroMyoGene Institute, INSERM U1217/CNRS UMR5310, Université Claude Bernard Lyon 1, Université de Lyon, Lyon, France

**Keywords:** IL-17A, IL-6, cytokine, autoimmune encephalitis, prognosis

## Abstract

**Introduction:**

Most of our knowledge into autoimmune encephalitis (AE) comes from N-Methyl-D-Aspartate Receptor (NMDAR) encephalitis. The concentrations of cytokines in cerebrospinal fluid (CSF) including IL-17A have been found to be increased and associated with poor outcome. However, data on the cytokine concentration in CSF and its correlation with outcome is lacking for other types of AE.

**Objective:**

To report the concentrations of CSF sIL-2R, IL-6, IL-8, IL-10 and IL-17A and to correlate it with acute disease severity and the 1-year outcome in non-NMDAR AE.

**Methods:**

We measured the CSF concentration of each cytokine in 20 AE patients, and compared IL-6 and IL-17A concentrations with 13 patients with CNS demyelinating diseases and 20 non-inflammatory controls. Patients were > 18yr and had at least 1-year clinical follow-up. Intracellular and NMDAR antibody (Ab) -mediated encephalitis were excluded. A mRS ≤ 2 was retained as a 1-year good outcome.

**Results:**

The IL-17A concentration in CSF was higher in AE patients than in both control groups (*p*<0.01). No difference was observed in CSF concentration of IL-6 between groups. At disease onset, a high CSF IL-17A concentration correlated with a high modified Rankin Scale (*p*<0.05), a high Clinical Assessment Scale for Autoimmune Encephalitis score (*p*<0.001) and ICU admission (*p*<0.01). There was no correlation between the concentration of all CSF cytokines and the 1-year clinical outcome.

**Conclusion:**

Our results show that CSF IL-17A could be interesting to assess initial severity in non-NMDAR AE. Thus, CSF IL-17A could be an interesting therapeutic target and be useful to assess early selective immunosuppressive therapy.

## Introduction

Autoimmune encephalitis (AE) is a non-infectious, immune-mediated central nervous system (CNS) disorder that mimics infectious encephalitis. It can be paraneoplastic from a cancer, or primarily auto-immune and result from cell- or antibody-mediated immune attack against either intracellular or cell surface antigens (1). It causes immune-mediated neural damage that can lead to different clinical presentations, from isolated cognitive impairment and behavioral changes to the life threatening status epilepticus. It must be associated with either a new focal CNS finding, CSF pleiocytosis, or MRI features suggestive of encephalitis. Importantly, patients can present without fever and a normal CSF associated with a normal MRI so the classical definition of AE may not apply. The different clinical presentations are thought to be associated with the different neuronal cell surface antibodies (Ab) (1–5). Most of our knowledge about AE comes from N-Methyl-D-Aspartate Receptor (NMDAR) encephalitis, which is the most frequent and most studied immune-mediated encephalitis worldwide. The outcome of patients with AE has gradually improved with the development of first-line immunotherapies, which includes steroids, intravenous immune globulins (IVIg), plasma exchange, or second-line treatment with rituximab and cyclophosphamide (6, 7) but several patients remain with refractory disease. Patient outcomes are related to the rapidity of administration and efficacy of immune treatment (6, 8). However, because of the difficulties related to the diagnostic work-up, therapeutic upgrade can take weeks and impact on prognosis. Early diagnosis is important and can be made prior to the Ab-test results as well as in the absence of positive autoimmune antibody detection (9). Specific tools have been developed to predict the 1-year outcome of patients presenting with NMDAR encephalitis (8, 10), which allow specific therapeutic management with induction or escalation strategies. There is no validated tool to predict outcome in other Ab-mediated AE. It is therefore important to find markers to predict the short- and long-term outcome of patients with other cell surface Ab-mediated AE.

Cytokines are low molecular weight proteins that mediate and regulate cells of the immune system. The dysregulation of cytokines plays a central role in the development of autoimmune disorders, including CNS autoimmune diseases such as multiple sclerosis (MS) (11) or neuromyelitis optica spectrum disorders (NMOSD) (12). Moreover, cytokine targeted therapies are now widely used to treat autoimmune diseases. Most of the studies investigating cytokines in AE have been performed on patients with NMDAR encephalitis (13–16). The CSF level of interleukin (IL)-17A has been shown to correlate with poor prognosis in this population (16, 17). However, only a few studies have focused on the role of cytokines in other types of AE. Knowledge into the role of cytokines in AE mediated by cell surface Ab has grown with the reported efficacy of IL-6R blockade in adult and pediatric cohorts of refractory AE (18, 19), which is suggestive of a key role of IL-6 during the inflammatory process. Taken together, these data suggest that cytokines could be interesting biomarkers that could indicate prognosis in AE patients.

Therefore, in the present study, we aimed to investigate the level of 5 selected cytokines (sIL-2R, IL-6, IL-8, IL-10 and IL-17A) in the CSF of a cohort of patients with non-NMDAR AE and to correlate their concentrations with patients outcomes.

## Materials and Methods

### Patients

Seventeen patients diagnosed with definite autoimmune limbic encephalitis (LE) according to the criteria of Graus et al. (9) and 3 patients with other types of seropositive autoimmune encephalitis presenting at the Neurology Department of the University Hospital of Nice, France, were included from January 31, 2014, until January 31, 2020. The inclusion criteria were based on: (i) an age over 18 years-old, (ii) a clinical follow-up of at least 12 months and (iii) enough frozen CSF to perform a cytokine analysis. Patients who died in the first year of follow-up were also included. Patients were excluded if they had: (i) intracellular neuronal Ab and/or NMDAR Ab and/or (ii) a CSF sampling obtained under any immunotherapy. Autoimmune encephalitis patients were recorded as follows: 10 anti-LGI1 LE, 2 anti-GABA_B_R LE, 2 anti-IgLON5 diseases, 1 anti-GABA_A_R LE, 1 anti-CASPR2 LE, 1 anti-AK5 LE, 2 seronegative LE, and 1 undefined neuronal cell surface Ab associated immune cerebellar ataxia. For all patients, neuronal cell surface Ab were identified using a cell based assay performed at the French Reference Center for Paraneoplastic Neurological Syndromes and Autoimmune Encephalitis, Hospices Civils de Lyon, Hôpital Neurologique, Lyon, France. Clinical data were pooled using the Clinical Assessment Scale for Autoimmune Encephalitis (CASE) (20). We also recorded the initial modified Ranking Scale (mRS), neoplasm status and Intensive Care Unit (ICU) admission for all patients. Brain MRI, CSF and treatment data were also gathered. The blood neutrophil to lymphocyte ratio (NLR) was calculated for each patient as a potential prognostic marker (21). The final outcome was determined using the mRS scoring at the 1-year follow-up visit for each patient. A mRS scoring ≤ 2 was retained as a good outcome whereas mRS scoring > 2 identified a poor outcome.

To assess CSF IL-6 and IL-17A expression in AE patients, we enrolled 20 age- and sex-matched patients with non-inflammatory neurological disease (NIND) as symptomatic negative controls and 13 sex-matched patients with CNS demyelinating diseases (multiple sclerosis and related disorders-MSARD) as inflammatory controls. Symptomatic control patients had idiopathic intracranial hypertension (IIH) (*n*=12) and cerebral vascular leukopathy (*n*=8).

### CSF Cytokine Measurement

CSF was collected during the diagnostic work-up during active disease for all patients and prior to immunosuppressive medication for AE and MSARD patients. The CSF was aliquoted within 2 hours of sampling, and kept frozen at -80°C until assayed.

Interleukin-6 and IL-10 were measured in routine using the LUMINEX (BioRad) platform and analysis was made according to the manufacturer’s instructions.

Soluble IL-2R, IL-8 and IL-17A were measured by enzyme-linked immunosorbent assay (ELISA) using kits: a human sIL-2R/CD25 kit, cat. No. BMS212-2 (Thermo Fischer Scientific) for sIL-2R, a human IL-8 Instant ELISA kit, cat. no. BMS204-3INST (Thermo Fischer Scientific) for IL-8 and a FIDIS Human IL17 ELISA Ready-SET-go (Ebiosciences; ref 88-7476-86) for IL-17A. Standards were reconstituted in assay diluents and serial dilutions were made to obtain a standard curve.

### Statistical Analysis

All analyses were made using MedCalc Statistical Software version 19.3.1.

Quantitative values were expressed as medians with their interquartile range (IQR) and qualitative data were expressed as percentages. The comparison of medians was performed using a Mann and Whitney or a Kruskal-Wallis test according to the number of groups to be compared. The correlation assessment between two quantitative data sets was done using a rank correlation test (Spearman’s rho). A p value of less than 0.05 was considered to be statistically significant.

## Results

### Baseline Characteristics of AE Patients

Autoimmune encephalitis patients were sex-matched with both controls, with a female to male ratio of 0.45, 0.46 and 0.40 (*p*=0.93) respectively in the AE, MSARD and NIND groups. However, the age-match was not met between the AE and MSARD groups with a median age of 67.7 and 54 years-old (*p*=0.012), respectively (**Table 1**).

**Table 1:** Baseline characteristics of AE patients.

	AE (n=20)	MSARD (n=13)	NIND (n=20)	P value
**Demographic data** Median age, years Female to Male ratio, % Death, n (%)	67.7 ± 9.1 45 3 (15)	54 ± 6 46 n/a	63.5 ± 13.2 40 n/a	<0.05 0.93
**Clinical data** Seizure, n (%) Altered consciousness, n (%) Abnormal behavior, n (%) Memory deficit, n (%) Movement disorder, n (%) Sleep disorder, n (%) Median mRS Median CASE ICU admission, n (%) Active neoplasm, n (%)	13 (65) 6 (30) 15 (75) 18 (90) 6 (30) 2 (10) 3 ± 0.25 9 ± 2.25 5 (25) 2 SCLC (10)	n/a n/a n/a n/a n/a n/a n/a n/a n/a n/a	n/a n/a n/a n/a n/a n/a n/a n/a n/a n/a	
**Biological data** Median NLR Median CSF protein (g/L), [range] Median CSF WBC count (/µL), [range] Median IgG index, [range] CSF OCBs, n (%) Antibody type Anti-LGI1 Ab, n (%) Other Ab subtype, n (%) Seronegative LE, n (%)	4.2 ± 2.6 0.41 [0.2 – 1.39] 1 [0 – 181] 0.62 [0.42 – 1.57] 4 (20) 10 (50) 8 (40) 2 (10)	2.1 ± 1.4 0.37 [0.2 – 0.61] 2 [0 – 12] 0.92 [0.55 – 1.99] 9 (69) n/a n/a n/a	1.8 ± 1.1 0.32 [0.11 – 0.62] 0 [0 – 3] 0.56 [0.41 – 0.77] 0 (0) n/a n/a n/a	<0.001 0.40 <0.01 <0.001 <0.0001
**MRI data** Specific T2 hyperintensities, n (%)	15 (75)	n/a	n/a	
**Treatment data** First line treatment, n (%) Steroids, n (%) IVIg, n (%) Plasma exchange, n (%) Second line treatment, n (%) Rituximab, n (%) Cyclophosphamide, n (%)	20 (100) 7 (35) 17 (85) 0 (0) 15 (75) 7 (35) 10 (50)	n/a n/a n/a n/a n/a n/a n/a	n/a n/a n/a n/a n/a n/a n/a	

AE, autoimmune encephalitis; MSARD, multiple sclerosis and related disorders; NIND, non inflammatory neurological disease; NLR, serum neutrophil to lymphocyte ratio; OCBs, oligoclonal bands; IVIg, intravenous immune globulins; SCLC, small cell lung cancer; Other Ab subtype, 2 anti-GABA_B_R Ab, 2 anti-IgLON5 Ab, 1 anti-GABA_A_R Ab, 1 anti-CASPR2 Ab; 1 anti-AK5 Ab and 1 Undefined neuronal cell surface Ab.

The patients mostly presented with a memory deficit (90%), abnormal behavior (75%) and seizures (65%) which are the most common clinical manifestations of LE. Three patients died during follow-up at 3, 9 and 15 months and had anti-IgLON5 disease, paraneoplastic undefined cell surface Ab associated cerebellar ataxia and paraneoplastic anti-GABA_B_R LE respectively. Six patients had altered consciousness and 5 were admitted into the ICU during their first hospital stay because of new onset refractory status epilepticus (NORSE, *n*=4) or severe altered consciousness (*n*=1). The median mRS score was 3 ± 0.25 (range from 2 to 5) and median CASE score was 9 ± 2.25 (range from 4 to 22). Fifteen patients presented with typical bilateral temporal T2 hyper intensities suggestive of LE on brain MRI. Only five patients presented with an inflammatory CSF: (i) 3 of them had an elevated CSF WBC count (> 10 cells/µL), (ii) all of them had an IgG index > 0.7 and (iii) 4 had CSF oligoclonal bands. All patients were treated with first-line immunotherapy (3 patients received steroids, 13 received IVIg and 4 received both) and 75% were treated with immunosuppressants in first-line (10 with cyclophosphamide and 5 with rituximab). Two patients relapse on cyclophosphamide within the first 6 months of treatment and were treated adding rituximab. At the 1-year follow-up visit time point, 8 patients were still with a mRS score > 2.

### Patients With Autoimmune Encephalitis Expressed a Higher CSF IL-17A Level Than Controls

To assess the pro-inflammatory cytokine profile of AE patients, we compared the CSF IL-6 and IL-17A concentrations between the AE, MSARD and NIND patients.

**Figure 1:**
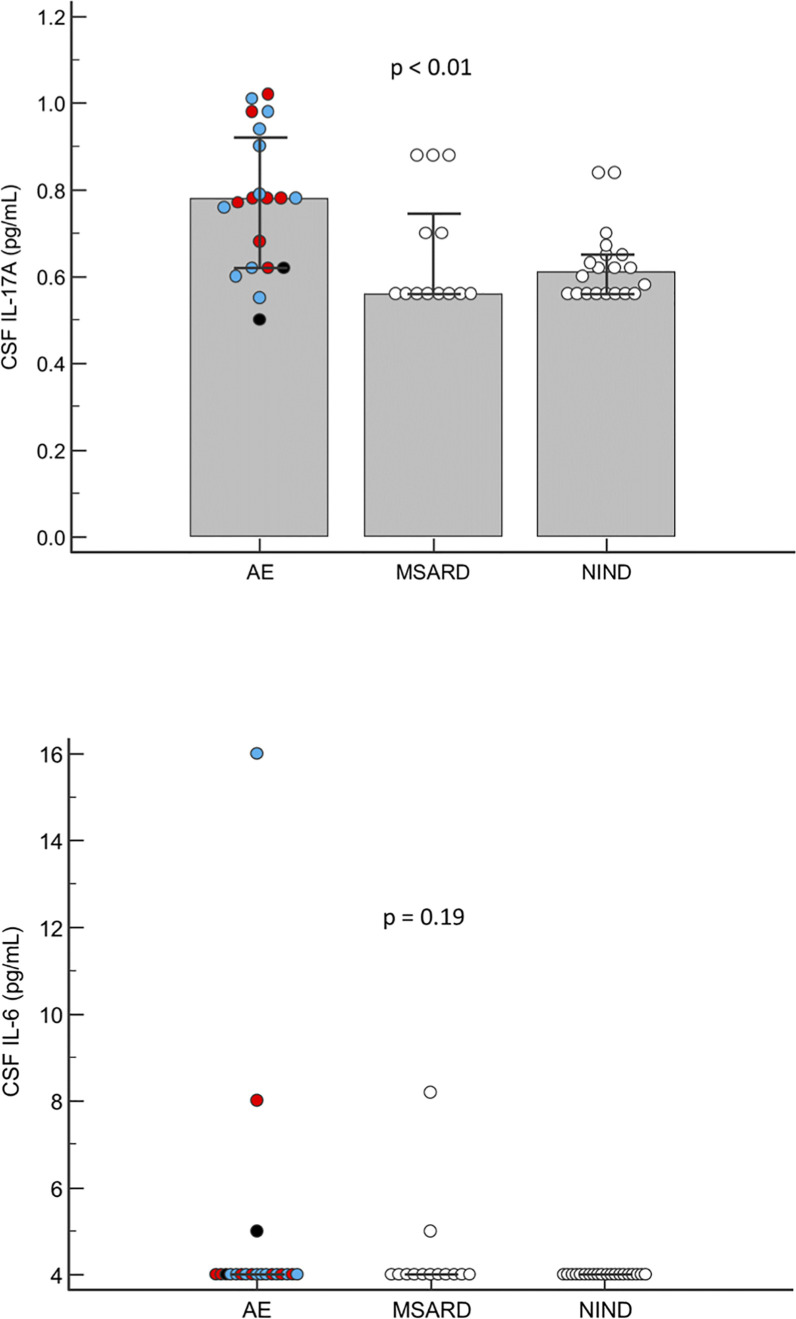
Cerebrospinal fluid profiles of IL-17A and IL-6 in AE patients compared to inflammatory (MSARD) and non-inflammatory (NIND) controls. Numbers of patients in each group are: AE group (*n*=20), MSARD group (*n*=13) and NIND group (*n*=20). Results are expressed as medians (Kruskal-Wallis test). The blue dots correspond to anti-LGI1 LE, the red dots to other cell-surface Ab mediated AE and the black dots to seronegative LE.

The CSF IL-17A concentration was higher in AE patients (median level of 0.78 pg/mL) than in both control groups (median of 0.56 and 0.61 pg/mL in the MSARD and NIND groups respectively, *p*=0.0088) (**Figure 1**). There was no difference in expression of CSF IL-17A between anti-LGI1 LE and the other subtypes of AE (median level of 0.78 and 0.79 pg/mL, respectively, *p*=0.62).

There was no difference in the CSF IL-6 concentration between the AE and control groups (*p*=0.19) (**Figure 1**) mainly because the CSF IL-6 level was rarely elevated in AE and MSARD groups (3 and 2 patients, respectively).

### CSF IL-17A Expression Correlated With Severe Illness at Onset

To determine if the CSF sIL-2R, IL-6, IL-8, IL-10 and IL-17A levels were associated with disease severity at onset in AE patients, we looked for a correlation between the concentrations of each CSF cytokine and (i) initial mRS, (ii) initial CASE scoring (both calculated within the first 48 hours of hospitalization) and (iii) admission to the ICU during the first hospitalization.

Cerebrospinal fluid IL-10 concentration was below the lower detection limit (5 pg/mL) for all AE patients.

There was no significant correlation between the mRS score and CSF sIL-2R concentration (median of 14, 11, 21 and 32 pg/mL for mRS group 2, 3, 4 and 5 respectively, *p*=0.70), CSF IL-6 concentration (median of 4 pg/mL for each mRS groups, *p*=0.15) and CSF IL-8 concentration (median of 91, 52, 34 and 49 pg/mL for mRS group 2, 3, 4 and 5 respectively, *p*=0.47). No correlation was found between the CASE score and CSF sIL-2R (*r*=0.08, *p*=0.73), CSF IL-6 (*r*=0.33, *p*=0.22) and CSF IL-8 (*r*=0.41, *p*=0.19) and neither between ICU admission and CSF sIL-2R (median of 32 vs 14 pg/mL for admitted patients vs non admitted, p=0.29), CSF IL-6 (median of 10 vs 4 pg/mL for admitted patients vs non admitted, p=0.09) and CSF IL-8 (median of 49 vs 54 pg/mL for admitted patients vs non admitted, p=0.80).

**Figure 2:**
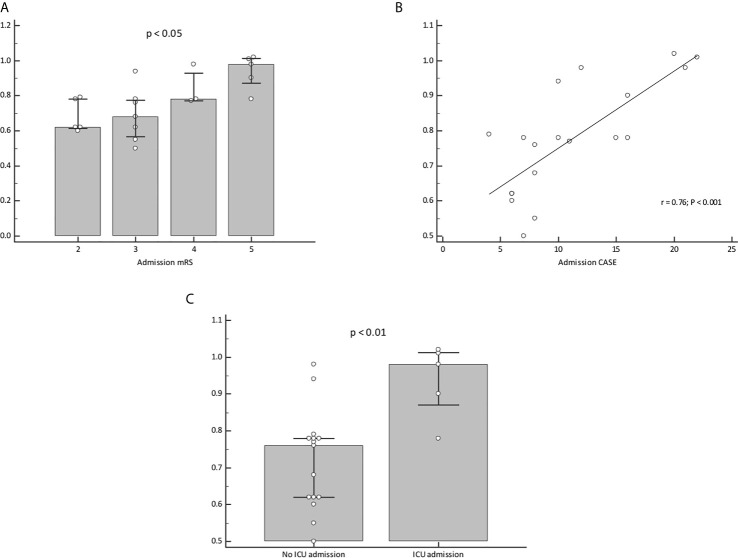
Cerebrospinal fluid IL-17A is associated with severe illness at onset in AE patients. Cerebrospinal fluid IL-17A correlate with the initial mRS score **(A)** and initial CASE score **(B)**. ICU admitted patients expressed higher level of CSF IL-17A then patients without ICU admission **(C)**.

However, the CSF IL-17A level correlated positively with mRS (median CSF IL-17A of 0.98, 0.78, 0.68 and 0.62 pg/mL for mRS group 5, 4, 3 and 2, respectively, *p*=0.028, **Figure 2A**) and CASE scoring (*r*=0.76, *p*<0.001) (**Figure 2B**). Cerebrospinal fluid IL-17A was also associated with ICU admission (median IL-17A of 0.98 vs 0.76 pg/mL, *p*=0.0074) (**Figure 2C**).

Most of the patients were admitted to the ICU because of NORSE. However, the CSF IL-17A concentration was not associated with a status epilepticus (median IL-17A of 0.77 vs 0.78 pg/mL in patients with and without seizures, respectively, *p*=0.52). There was no correlation between CSF IL-17A and other sub items of the CASE score while CSF IL-17A did not correlate with psychiatric symptoms (p=0.79), dystonia/dyskinesia (p=0.18) or memory dysfunction (19/20 patients presented at admission with severe memory deficits, p not available). The only sub item that correlate with CSF IL-17A was altered consciousness (p<0.01) directly linked to ICU admission.

### CSF Cytokines Concentration at Disease Onset Could Not Predict the One-Year Outcome

To determine if the concentrations of CSF cytokines correlated with the 1-year outcome, we separated the AE patients into 2 groups according to their mRS scoring at the 1-year follow-up visit. There was no association between CSF cytokines levels and the 1-year clinical outcome: median CSF IL-2R concentration of 13 vs 21 pg/mL, *p*=0.85; median CSF IL-6 concentration of 4 pg/mL for both groups, *p*=0.88; median CSF IL-8 concentration of 78 vs 40 pg/mL, *p*=0.18 and median CSF IL-17A concentration of 0.78 vs 0.70 pg/mL, *p*=0.67 for patients with a 1-year good outcome vs patients with a bad outcome respectively. The only factor that could predict the 1-year outcome in our AE population was treatment delay with a median of 23 days in the good outcome group vs 46 days in the poor outcome group (*p*=0.14). However, the difference between both groups was not statistically different.

## Discussion

The present study shows that the concentration of CSF IL-17A was increased in non-NMDAR cell surface Ab encephalitis and that this increase was associated with disease severity at onset. However, no correlation was found between the CSF IL17-A (or other studied cytokines) concentration and the 1-year outcome. This could be explained by the early management with immunosuppressive treatment of our patients: 15/20 patients were treated with rituximab or cyclophosphamide as first-line therapy. However, even being not statistically significant, we found that treatment delay could be an important 1-year outcome predicting factor. This result is consistent with those reported in 2 large series of NMDAR (8, 10). Other studies also found that an early immunosuppressive treatment could prevent long-term cognitive deficits in anti-LGI-1 LE (22, 23). These findings support the need to assess early diagnosis of AE patients in order to initiate specific treatment as soon as possible.

Interleukin-17A, the main IL of the IL-17 family, is a pro-inflammatory cytokine secreted by type 17 helper T cells (Th17 cells). It is involved in the inflammatory response to extracellular bacterial or fungal infections (24). It plays a central role in chronic inflammation by: (i) increasing the neutrophil count by G-CSF stimulation, (ii) attracting neutrophils to the inflammatory site by the production of chemokines such as IL-8, (iii) maintaining the inflammatory process by the secretion of pro-inflammatory cytokines related to macrophage stimulation (cf. IL-1β, IL-6 and TNF-α) and promoting CCL20 production, allowing the chemotaxis of Th17 cells (25, 26).

The role of IL-17A in many autoimmune diseases is now well-known. In neurology, IL-17A has been found to be increased in MS and related disorders but also in NMDAR encephalitis and refractory epilepsy (RE) (15, 27). Kumar et al. reported a similar immune profile between children with RE and Rasmussen’s encephalitis with expansion of CD4+ IL17+ T cells and expansion of CD25+ B cells suggesting that both Th17 and B cells are involved in the pathogenesis of RE and AE (27). However, epilepsy in patients with NIND does not result in a high level of CSF IL-17A or IL-6 as compared to immune-mediated epilepsy in the elderly (28). In our study, there was no difference in the CSF IL-17A concentration between AE patients with and without seizures. However, 4 out of the 5 ICU admitted AE patients had RE, suggesting that the CSF IL-17A concentration could be a key factor in the development of NORSE.

Our results are consistent with previous published data suggesting a role of the Th17 pathway in CNS inflammation related to AE. An increase in both IL-17A and IL-6 in CSF was first found in small series of NMDAR encephalitis (15, 29). Zeng et al. found an increased number of Th17 CD4+ T cells in the CSF but not in serum of NMDAR encephalitis patients, suggesting intrathecal synthesis of IL-17A (17). Cerebrospinal fluid IL-17A has also been found to positively correlate with poor outcome in NMDAR encephalitis (16, 17). Taken together, these data suggest the role of CSF Th17 cells in the pathogenesis of NMDAR encephalitis.

To our knowledge, only a few studies have shown interest in cytokine detection in other types of AE than NMDAR encephalitis (15, 30, 31) and none of them looked for a correlation with AE outcome. Byun et al. reported a series of 10 anti-LGI1 LE, 14 NMDAR encephalitis and 10 NIND controls. They found an increased IL-17A concentration in CSF but not in serum of both NMDAR and anti-LGI1 LE patients. However, IL-6 was found to be increased only in the CSF of NMDAR encephalitis (15). Added to our results, this suggests that the Th17 pathway is associated with inflammation of the CNS in non-NMDAR AE. Knowing this, an elevated IL-17A in CSF at onset could be useful for early immunosuppressive management to prevent short-term outcome. In addition, the assay of such biomarkers requires only a few microliters of CSF and the results can be reported quickly.

Some AE patients may express elevated IL-6 in the CSF. Interestingly, we observed that 2 of the 3 patients with elevated CSF IL-6 presented with high mRS and CASE scores, and were both admitted to ICU at disease onset. However, the correlation between CSF IL-6 and severe illness at onset was not significant, maybe because of the small size of our cohort. It would be interesting to look at the IL-6 concentration in CSF in a larger cohort to better appreciate this phenotype.

Our study is the first to find a correlation between IL-17A in CSF and disease severity at onset in this AE population. These results tend toward expression of the Th17 inflammatory pathway that leads to inflammation of the CNS in neuronal cell surface Ab encephalitis. Nevertheless, this result must be confirmed in a larger cohort of patients.

We noted that the CSF IL-17A concentrations in our cohort were different to those reported in other studies. This is explained by the ELISA kit we used for analysis and the results are consistent with previously published ones (32). In our cohort, symptomatic controls expressed the same level of CSF IL-17A as MSARD patients. This could be explained by the fact that most of our negative controls suffered from idiopathic intracranial hypertension, patients known to express a high level of IL-17A in the CSF (32, 33).

To our knowledge, this is the largest series to date that looked at the CSF cytokine concentration in non-NMDAR AE, despite being a monocentric study with no more than 20 AE patients. We should point out that while we examined non-NMDAR encephalitis, the heterogeneity of Ab-mediated encephalitis in our cohort may be biased. Last but not least, our study lacks follow-up clinical and biological data. Being a retrospective study, we were not able to assess the CASE scoring at the one-year follow-up mark because of lacking clinical data. The CASE score, more specific to AE than the mRS, would probably be a better reflect of patients’ outcomes. As mentioned above, our results need to be confirmed with larger and more homogenized cohorts but nonetheless it reveals the fact that the Th17 pathway is implicated in non-NMDAR AE.

## Conclusion

The concentration of pro-inflammatory IL-17A is increased in the CSF of AE patients and correlates with disease severity at onset. Therefore, IL-17A in CSF could be useful to assess short-term severity and could lead to early immunosuppressive management for patients. Furthermore, the IgG1κ monoclonal antibody that binds to IL-17A could be an interesting alternative treatment for refractory cases.

## Data Availability Statement

The original contributions presented in the study are included in the article. Further inquiries can be directed to the corresponding author.

## Ethics Statement

The studies involving human participants were reviewed and approved by Institutional review board of the University Hospital of Nice. The patients/participants provided their written informed consent to participate in this study.

## Author Contributions

ML and CL-F designed the study, collected the data, performed the data analysis, and drafted the manuscript. VB, NC, and AD helped with collection of the study samples and clinical information. VB, JH, and PT helped with the manuscript preparation and the review for intellectual content. All authors contributed to the article and approved the submitted version.

## Conflict of Interest

The authors declare that the research was conducted in the absence of any commercial or financial relationships that could be construed as a potential conflict of interest.
